# Supplementary material for: Social and Economic Factors and Malnutrition or the Risk of Malnutrition in the Elderly: A Systematic Review and Meta-Analysis of Observational Studies
Source: Nutrients. 2020 Mar 11;12(3):737. doi: 10.3390/nu12030737 (PMC7146387; doi:10.3390/nu12030737)
Supplement: Supplementary file 1 [file nutrients-12-00737-s001.pdf]

Supplementary Files

Table S1. Preferred Reporting Items for Systematic Reviews and Meta-Analyses (PRISMA) criteria

**Section/topic**		**Checklist item**	**Page**
**TITLE**			
Title	1	Identify the report as a systematic review, meta-analysis, or both.	0
**ABSTRACT**			
Structured summary	2	Provide a structured summary including, as applicable: background; objectives; data sources; study eligibility criteria, participants, and interventions; study appraisal and synthesis methods; results; limitations; conclusions and implications of key findings; systematic review registration number.	2
**INTRODUCTION**			
Rationale	3	Describe the rationale for the review in the context of what is already known.	3-4
Objectives	4	Provide an explicit statement of questions being addressed with reference to participants, interventions, comparisons, outcomes, and study design (PICOS).	5
**METHODS**			
Protocol and registration	5	Indicate if a review protocol exists, if and where it can be accessed (e.g., Web address), and, if available, provide registration information including registration number.	6
Eligibility criteria	6	Specify study characteristics (e.g., PICOS, length of follow-up) and report characteristics (e.g., years considered, language, publication status) used as criteria for eligibility, giving rationale.	6
Information sources	7	Describe all information sources (e.g., databases with dates of coverage, contact with study authors to identify additional studies) in the search and date last searched.	6
Search	8	Present full electronic search strategy for at least one database, including any limits used, such that it could be repeated.	6
Study selection	9	State the process for selecting studies (i.e., screening, eligibility, included in systematic review, and, if applicable, included in the meta-analysis).	6-7
Data collection process	10	Describe method of data extraction from reports (e.g., piloted forms, independently, in duplicate) and any processes for obtaining and confirming data from investigators.	7
Data items	11	List and define all variables for which data were sought (e.g., PICOS, funding sources) and any assumptions and simplifications made.	7
Risk of bias in individual studies	12	Describe methods used for assessing risk of bias of individual studies (including specification of whether this was done at the study or outcome level), and how this information is to be used in any data synthesis.	7
Summary measures	13	State the principal summary measures (e.g., risk ratio, difference in means).	7
Synthesis of results	14	Describe the methods of handling data and combining results of studies, if done, including measures of consistency (e.g., I^2) for each meta-analysis.	7
Risk of bias across studies	15	Specify any assessment of risk of bias that may affect the cumulative evidence (e.g., publication bias, selective reporting within studies).	7
Additional analyses	16	Describe methods of additional analyses (e.g., sensitivity or subgroup analyses, meta-regression), if done, indicating which were pre-specified.	7
**RESULTS**			
Study selection	17	Give numbers of studies screened, assessed for eligibility, and included in the review, with reasons for exclusions at each stage, ideally with a flow diagram.	8
Study characteristics	18	For each study, present characteristics for which data were extracted (e.g., study size, PICOS, follow-up period) and provide the citations.	8-12
Risk of bias within studies	19	Present data on risk of bias of each study and, if available, any outcome level assessment (see item 12).	12

Results of individual studies	20	For all outcomes considered (benefits or harms), present, for each study: (a) simple summary data for each intervention group (b) effect estimates and confidence intervals, ideally with a forest plot.	13, 24-41
Synthesis of results	21	Present results of each meta-analysis done, including confidence intervals and measures of consistency.	12-14
Risk of bias across studies	22	Present results of any assessment of risk of bias across studies (see Item 15).	12, 42-43
Additional analysis	23	Give results of additional analyses, if done (e.g., sensitivity or subgroup analyses, meta-regression [see Item 16]).	13-14
**DISCUSSION**			
Summary of evidence	24	Summarize the main findings including the strength of evidence for each main outcome; consider their relevance to key groups (e.g., healthcare providers, users, and policy makers).	15-17
Limitations	25	Discuss limitations at study and outcome level (e.g., risk of bias), and at review-level (e.g., incomplete retrieval of identified research, reporting bias).	17-18
Conclusions	26	Provide a general interpretation of the results in the context of other evidence, and implications for future research.	18
**FUNDING**			
Funding	27	Describe sources of funding for the systematic review and other support (e.g., supply of data); role of funders for the systematic review.	-

Source: Moher D, Liberati A, Tetzlaff J, Altman DG, The PRISMA Group (2009). Preferred Reporting Items for Systematic Reviews and Meta-Analyses: The PRISMA Statement. PLoS Med 6(6): e1000097. DOI:10.1371/journal.pmed100009

Table S2. Characteristics of included studies about the relationship between educational level and malnutrition and/or malnutrition risk

Study	Type of study	Country or region	Number of participants	Age	Nutritional status assessment tool	Results	Having a low educational level is related to:	
							Risk of malnutrition or malnutrition	No mal-nutrition
**Significant studies**								
1. Donini, et al; 2013 [14]	Cross-sectional	Italy	n=718<br>M: 246<br>F: 472	≥ 65 years	MNA	RMN nursing home: illiterate 8.6%; elementary level 47.1%; medium-low level 28.6%; medium-high level 12.9%; degree 2.9%; p<0.05<br>RMN home: illiterate 19.2%; elementary level 60.8%; medium-low level 10.0%; medium-high level 6.9%; degree 3.1%; p<0.05<br>MN nursing home: illiterate 15.6%; elementary level 45.3%; medium-low level 20.3%; medium-high level 14.1%; degree 4.7%; p<0.05<br>MN home: illiterate 16.2%; elementary level 73.0%; medium-low level 8.1%; medium-high level 2.7%; degree 0.0%; p<0.05	×	
2. El-Desouky, et al; 2018 [45]	Cross-sectional	Egypt	n=320<br>M: 167<br>F: 153	≥ 60 years	MNA	<u>OR (95% CI):</u> lower than secondary level 3.93 (1.98-7.8) p<0.001	○	
3. Ferdous, et al; 2009 [41]	Cross-sectional	Bangladesh	n=457<br>M: 208<br>F: 249	≥ 60 years	MNA	<u>β coefficient:</u> literate 0.15 p=0.002	×	
4. Ferra, et al; 2012 [22]	Cross-sectional	Spain	n=402<br>M: 187<br>F: 215	≥ 65 years	BMI	<u>OR (95% CI):</u> low educational level 0.80 (0.42-1.54) p=0.024<br>Adjusted by gender and age.		○[a]
5. Gündüz, et al; 2015 [52]	Cross-sectional	Turkey	n=1,030<br>M: 464<br>F: 566	≥ 65 years	MNA	<u>OR (95% CI):</u> low educational level 0.568 (0.359-0.897) p<0.015		○
6. Jésus, et al; 2017 [46]	Cross-sectional	Central Africa	n=990<br>M: 406<br>F: 585	≥ 65 years	BMI	<u>OR (95% CI):</u> no formal education 1.4 (1.001-1.9) p=0.045	○	
7. Krzymińska-Siemaszko, et al; 2014 [39]	Cross-sectional	Poland	n=4,482<br>M: 2,340<br>F: 2,142	≥ 65 years	MNA	RMN: lower than primary level 55.26% (n=310); higher than primary level 40.69% (n=1,539); p<0.001<br>MN: lower than primary level 20.68% (n=116); higher than primary level 9.84% (n=372); p<0.001	×	

8. Lin, et al; 2016 [53]	Cross-sectional	China	n=708<br>M: 377<br>F: 371	≥ 60 years	MNA	MN: no education 35.2% (n=106); primary school 21.9% (n=65); middle school or above 10.0% (n=11); p<0.001<br>Optimal nutritional status: no education 64.8% (n=195); primary school 78.1% (n=232); middle school or above 90.0% (n=99); p<0.001	×	
9. Mathew, et al; 2016 [42]	Cross-sectional	India	n=190<br>M: 57<br>F: 133	≥ 60 years	MNA	OR (95% CI): schooling up to 12 years 8.064 p=0.044	○	
10. Mitri, et al; 2016 [25]	Cross-sectional	Lebanon	n=905<br>M: 533<br>F: 372	≥ 65 years	MNA	MN: low level (elementary/primary) 52.6% (n=320) p<0.001<br>Optimal nutritional status: low level (elementary/primary) 47.4% (n=288) p<0.001	×	
11. Mokhber, et al; 2011 [28]	Cross-sectional	Iran	n=1,565<br>M: 720<br>F: 845	≥ 60 years	MNA	RMN: no education 49.1%; education 25.9%; p<0.001<br>MN: no education 12.7%; education 7.6%; p<0.001	×	
12. Olayiwola, et al; 2006 [47]	Cross-sectional	Nigeria	n=305<br>M: 163<br>F: 142	≥ 60 years	NSI	Pearson correlation: r=0.25 p<0.05		×
13. Park, et al; 2014 [27]	Cross-sectional	Korea	n=15,146<br>M: 6,185<br>F: 8,961	≥ 60 years	NSI	MN: 0-6 years 46.9% (n=2,771) p=0.001<br>Optimal nutritional status: 0-6 years 53.1% (n=3,137) p=0.001		×
14. Ramage-Morin, et al; 2013 [49]	Cross-sectional	Canada	n=15,669<br>M: 6,334<br>F: 9,335	≥ 65 years	SCREEN II	OR (95% CI): lower than secondary level M: 1.31 (1.12-1.53) p<0.05, F: 1.42 (1.26-1.61) p<0.05	○	
15. Timpini, et al; 2011 [35]	Cross-sectional	Italy	n=698<br>M: 290<br>F: 408	≥ 65 years	MNA	OR (95% CI): schooling <5 years 2.7 (1.4-5.2) p<0.05	○	
**No significant studies**								
16. Bardon, et al; 2018 [19]	Cohort	Ireland	n=1,841<br>H: 916<br>N: 925	≥ 65 years	BMI and weight loss	OR (95% CI): secondary level 0.95 (0.67-1.36); primary level/no education 0.90 (0.63-1.28); p>0.05		
17. Cabrera, et al; 2007 [18]	Cross-sectional	Brazil	n=267<br>M: 107<br>F: 160	≥ 60 years	MNA	OR (95% CI): low educational level 2.17 (0.92-5.09) p=0.07		
18. Chen, et al; 2007 [16]	Cohort	Taiwan	n=114<br>M: 50<br>F: 64	≥ 65 years	MNA	MN: illiterate 22.7±3.8; sixth grade 23.9±3.0; ninth grade 23.9±2.0; >12 grade 22.3±4.8; p=0.23		

19. Damayanthi, et al; 2018 [29]	Cross-sectional	Sri Lanka	n=999<br>H: 251<br>M: 748	≥ 60 years	MNA	<u>OR (95% CI):</u> no education 1.98 (0.90-4.34) p=0.288; primary level 1.36 (0.85-2.17) p=0.297	
20. Debnath, et al; 2017 [44]	Cross-sectional	Bangladesh	n=330<br>H: 145<br>M: 185	≥ 60 years	IMC	MN: illiterate 49.4% (n=131); primary level 60.0% (n=15); higher than primary level 24.0% (n=6); p=0.051	
21. El Zoghbi, et al; 2014 [20]	Cross-sectional	Lebanon	n=111<br>H: 55<br>M: 56	≥ 65 years	MNA	MN: low educational level 57.4% (n=31) p=0.620	
22. Farre, et al; 2014 [26]	Cross-sectional	Spain	n=328<br>H: 126<br>M: 202	85 years	MNA	MN: schooling <6 years 84.1% (n=95) p=0.17	
23. Ji, et al; 2012 [37]	Cross-sectional	China	n=632<br>M: 208<br>F: 424	≥ 90 years	MNA	<u>Logistic regression:</u> no formal education -0.08 p=0.76	
24. Jun, et al; 2016 [51]	Cross-sectional	Shanghai	n=2,556<br>M: 0<br>F: 2,556	≥ 60 years	MNA	MN: schooling 0 years 33.0% (n=350); 1-9 years 47.4% (n=502); >9 years 19.6% (n=208); p=0.0680	
25. Madeira, et al; 2018 [24]	Cross-sectional	Portugal	n=1,186<br>H: 322<br>M: 864	≥ 65 years	MNA	<u>OR (95% CI):</u> schooling >5 years 0.62 (0.29-1.35); p>0.05	
26. Maseda, et al; 2017 [13]	Cross-sectional	Spain	n=749<br>H: 295<br>M: 454	≥ 65 years	MNA	<u>Pearson correlation</u>: education years r=0.042 p>0.05	
27. Naidoo, et al; 2015 [43]	Cross-sectional	South Africa	n=984<br>H: 224<br>M: 760	≥ 60 years	MNA	RMN: no education 24.1% (n=103); primary level 44.3% (n=189); secondary level 29.5% (n=126); tertiary level 2.1% (n=9); p=0.430<br>MN: no education 25.9% (n=14); primary level 31.5% (n=17); secondary level 40.7% (n=22); tertiary level 1.9% (n=1); p=0.430	
28. Schilp, et al; 2011 [33]	Cohort	Amsterdam	n=1,120<br>M: 577<br>F: 543	≥ 65 years	BMI	<u>HR (95% CI):</u> low level Ref.; medium level 0.78 (0.56-1.09); high level 0.94 (0.56-1.58); p>0.05	
29. Shi, et al; 2014 [30]	Cross-sectional	China	n=558<br>H: 245<br>M: 313	≥ 60 years	MNA	MN: illiterate (0 years) 9.5% (n=12); low level (1-9 years) 43.7% (n=55); high level (>9 years) 46.8% (n=59); p=0.057	

30. Suzana, et al; 2013 [36]	Cross-sectional	Malaysia	n=160<br>M: 58<br>F: 102	≥ 60 years	MNA	RMN: no education 50.0% (n=12) p=0.420<br>Optimal nutritional status: no education 50.0% (n=12) p=0.420	
31. Wham, et al; 2015 [48]	Cross-sectional	New Zealand	n=67<br>H: 30<br>M: 37	≥ 75 years	SCREEN II	MN: primary level 44.9±3.72; secondary level 47.5±5.04; tertiary level 49.2±5.90; p=0.15	

M: Male; F: Female; MN: Malnutrition; RMN: Risk of malnutrition; MNA: Mini Nutritional Assessment; BMI: Body Mass Index; SCREEN II: Senior in the community: risk evaluation for eating and nutrition; NSI: Nutritional Screening Initiative; OR: Odds Ratio; HR: Hazard Ratio; ○: Based on Odds Ratio; ×: Not based on Odds Ratio

[a]Odds Ratio obtained from the low weight evaluated by the Body Mass Index

Table S3. Characteristics of included studies about the relationship between living alone or not and malnutrition and/or malnutrition risk

Study	Type of study	Country or region	Number of participants	Age	Nutritional status assessment tool	Results	Living alone is related to: Risk of malnutrition or malnutrition	No mal-nutrition
**Significant studies**								
1. Donini, et al; 2013 [14]	Cross-sectional	Italy	n=718<br>M: 246<br>F: 472	≥ 65 years	MNA	RMN nursing home: living alone 32.3%; spouse 41.9%; children 9.7%; grandchildren 3.2%; friends 1.6%; caregiver 11.3%; p<0.05<br>RMN home: living alone 17.1%; spouse 48.1%; children 25.6%; grandchildren 0.8%; friends 2.3%; caregiver 6.2%; p<0.05<br>MN nursing home: living home 43.4%; spouse 24.5%; children 11.3%; grandchildren 3.8%; friends 1.9%; caregiver 15.1%; p<0.05<br>MN home: living alone 2.7%; spouse 40.5%; children 45.9%; grandchildren 2.7%; friends 5.4%; caregiver 2.7%; p<0.05	×[b]	
2. El-Desouky, et al; 2018 [45]	Cross-sectional	Egypt	n=320<br>M: 167<br>F: 153	≥ 60 years	MNA	MN: living alone 24.7% (n=58); cohabit 75.3% (n=177); p<0.001		×
3. Eskelinen, et al; 2016 [21]	Cross-sectional	Finland	n=573<br>M: 171<br>F: 402	≥ 75 years	MNA	MN: living alone 65.0% (n=119) p=0.005	×	
4. Jun, et al; 2016 [51]	Cross-sectional	Shanghai	n=2,556<br>M: 0<br>F: 2,556	≥ 60 years	MNA	MN: cohabit 51.7% (n=548); living alone 48.3% (n=512); p<0.0001		×
5. Krzymińska-Siemaszko, et al; 2014 [39]	Cross-sectional	Poland	n=4,482<br>M: 2,340<br>F: 2,142	≥ 65 years	MNA	RMN: living alone 44.77% (n=424); cohabit 41.82% (n=1,406); p<0.001<br>MN: living alone 12.57% (n=119); cohabit 10.80% (n=363); p<0.001	×	
6. Lengyel, et al; 2017 [50]	Cohort	Canada	n=336<br>M: 336<br>F: 0	90 years	SCREEN II	RMN: living alone: RMN high-increased 60.0%; RMN high 49.3%; RMN medium-increased 38.8%; RMN medium 20.5%; RMN low 14.3%; p<0.001	×	
7. Lin, et al; 2016 [53]	Cross-sectional	China	n=708<br>M: 377<br>F: 371	≥ 60 years	MNA	MN: living alone 40.9% (n=52); live with the spouse 23.2% (n=86); live with children 20.9% (n=44); p<0.001	×	

8. Maseda, et al; 2017 [13]	Cross-sectional	Spain	n=749<br>M: 295<br>F: 454	≥ 65 years	MNA	MN: live with the spouse 46.7% (n=50) p=0.016<br>Optimal nutritional status: live with the spouse 59.1% (n=379) p=0.016	×
9. Mokhber, et al; 2011 [28]	Cross-sectional	Iran	n=1,565<br>M: 720<br>F: 845	≥ 60 years	MNA	RMN: living alone 52.8%; cohabit 53.8%; with family 39.4%; p<0.001<br>MN: living alone 14.4%; cohabit 14.0%; with family 10.3%; p<0.001	×
10. Park, et al; 2014 [27]	Cross-sectional	Korea	n=15,146<br>M: 6,185<br>F: 8,961	≥ 60 years	NSI	MN: living alone 73.5% (n=2,559) p=0.001<br>Optimal nutritional status: living alone 26.5% (n=925) p=0.001	×
11. Ramage-Morin, et al; 2013 [49]	Cross-sectional	Canada	n=15,669<br>M: 6,334<br>F: 9,335	≥ 65 years	SCREEN II	<u>OR (95% CI):</u> M: living alone 3.19 (2.73-3.73) p<0.05; F: living alone 2.01 (1.78-2.27) p<0.05	○
12. Rodriguez-Tadeo, et al; 2012 [32]	Cross-sectional	Mexico	n=760<br>M: 190<br>F: 570	≥ 60 years	MNA	<u>OR (95% CI):</u> living alone 1.762 (1.307-2.376) p=0.000<br><u>β coefficient:</u> living alone 0.126 p=0.001	○
13. Westergren, et al; 2014 [38]	Cross-sectional	Sweden	n=465<br>M: 216<br>F: 249	≥ 70 years	SCREEN II	<u>OR (95% CI):</u> woman lives alone 4.63 (2.85-7.52) p<0.001; man lives alone 6.23 (3.35-11.59) p<0.001	○
**No significant studies**							
14. Bardon, et al; 2018 [19]	Cohort	Ireland	n=1,841<br>M: 916<br>F: 925	≥ 65 years	BMI and weight loss	<u>OR (95% CI):</u> living alone 1.21 (0.88-1.65); p>0.05	
15. Damayanthi, et al; 2018 [29]	Cross-sectional	Sri Lanka	n=999<br>M: 251<br>F: 748	≥ 60 years	MNA	<u>OR (95% CI):</u> living alone 1.25 (0.24-6.42) p=0.782	
16. Debnath, et al; 2017 [44]	Cross-sectional	Bangladesh	n=330<br>M: 145<br>F: 185	≥ 60 years	BMI	MN: live with children 46.9% (n=122); with wife 51.4% (n=19); alone 61.1% (n=11); p=0.779	
17. Farre, et al; 2014 [26]	Cross-sectional	Spain	n=328<br>M: 126<br>F: 202	85 years	MNA	MN: living alone 62.5% (n=30) p=0.31	
18. Ferra, et al; 2012 [22]	Cross-sectional	Spain	n=402<br>M: 187<br>F: 215	≥ 65 years	BMI	<u>OR (95% CI):</u> living alone 0.67 (0.29-1.54) p=0.303[a]	

19. Gündüz, et al; 2015 [52]	Cross-sectional	Turkey	n=1,030<br>M: 464<br>F: 566	≥ 65 years	MNA	RMN: live with the spouse 73.3% (n=220); alone 9.3% (n=28); children 15.7% (n=47); family 1.7% (n=5); p=0.24<br>MN: live with the spouse 70.4% (n=138); alone 7.1% (n=14); children 18.4% (n=36); family 4.1% (n=8); p=0.24	
20. Ji, et al; 2012 [37]	Cross-sectional	China	n=632<br>M: 208<br>F: 424	≥ 90 years	MNA	Logistic regression: living alone -0.14 p=0.68	
21. Johansson, et al; 2009 [40]	Cross-sectional	Sweden	n=258<br>M: 128<br>F: 130	71-80 years	MNA	RMN: living alone H: 45%; M: 81%; p=UA	
22. Mathew, et al; 2016 [42]	Cross-sectional	India	n=190<br>M: 57<br>F: 133	≥ 60 years	MNA	OR (95% CI): living alone 0.817 p=0.800	
23. Mitri, et al; 2016 [25]	Cross-sectional	Lebanon	n=905<br>M: 533<br>F: 372	≥ 65 years	MNA	MN: living alone 48.4% (n=75) p=0.978<br>Optimal nutritional status: living alone 51.6% (n=80) p=0.978	
24. Naidoo, et al; 2015 [43]	Cross-sectional	South Africa	n=984<br>M: 224<br>F: 760	≥ 60 years	MNA	RMN: house with >5 people 53.8% (n=229) p=0.301; house with grandchildren 73.8% (n=315) p=0.740<br>MN: house with >5 people 53.7% (n=29) p=0.301; house with grandchildren 70.4% (n=38) p=0.740	
25. Romero-Ortuno, et al; 2011 [31]	Cross-sectional	Ireland	n=556<br>M: ND<br>F: ND	≥ 60 years	MNA	OR (95% CI): living alone 1.520 (0.718-3.218) p=0.274<br>β coefficient: living alone -0.419 p=0.274	
26. Suzana, et al; 2013 [36]	Cross-sectional	Malaysia	n=160<br>M: 58<br>F: 102	≥ 60 years	MNA	RMN: living alone 46.2% (n=6) p=0.781<br>Optimal nutritional status: living alone 53.8% (n=7) p=0.781	
27. Wham, et al; 2015 [48]	Cross-sectional	New Zealand	n=67<br>M: 30<br>F: 37	≥ 75 years	SCREEN II	MN: living alone 46.3±5.53; cohabit 48.5±5.15; p=0.12	

M: Male; F: Female; MN: Malnutrition; RMN: Risk of malnutrition; MNA: Mini Nutritional Assessment; BMI: Body Mass Index; SCREEN II: Senior in the community: risk evaluation for eating and nutrition; NSI: Nutritional Screening Initiative; UA: Unavailable; OR: Odds Ratio; ○: Based on Odds Ratio; ×: Not based on Odds Ratio

[b]Only relation with malnutrition was established in the nursing home

Table S4. Characteristics of included studies about the relationship between marital status and malnutrition and/or malnutrition risk

Study	Type of study	Country or region	Number of participants	Age	Nutritional status assessment tool	Results	Be single, widowed or divorced is related to:	
							Risk of malnutrition or malnutrition	No mal-nutrition
**Significant studies**								
1. Bardon, et al; 2018 [19]	Cohort	Ireland	n=1,841<br>M: 916<br>F: 925	≥ 65 years	BMI and weight loss	OR (95% CI): not married/divorced 1.64 (1.09-2.47); p<0.05	○	
2. Donini, et al; 2013 [14]	Cross-sectional	Italy	n=718<br>M: 246<br>F: 472	≥ 65 years	MNA	RMN nursing home: single 14.5%; married 43.5%; widowed 36.2%; divorced 5.8%; p<0.05<br>RMN home: single 10.0%; married 52.3%; widowed 36.9%; divorced 0.8%; p<0.05<br>MN nursing home: single 22.6%; married 29.0%; widowed 48.4%; divorced 0.0%; p<0.05<br>MN home: single 8.1%; married 48.6%; widowed 43.2%; divorced 0.0%; p<0.05	×[b]	
3. El-Desouky, et al; 2018 [45]	Cross-sectional	Egypt	n=320<br>M: 167<br>F: 153	≥ 60 years	MNA	OR (95% CI): divorced/widowed 29.4 (8.9-94.4) p<0.001	○	
4. Krzymińska-Siemaszko, et al; 2014 [39]	Cross-sectional	Poland	n=4,482<br>M: 2,340<br>F: 2,142	≥ 65 years	MNA	RMN: not married 47.56% (n=1,014); married 37.79% (n=834); p<0.001<br>MN: not married 16.14% (n=344); married 6.57% (n=145); p<0.001	×	
5. Lin, et al; 2016 [53]	Cross-sectional	China	n=708<br>M: 377<br>F: 371	≥ 60 years	MNA	MN: single 49.2% (n=58); married 22.4% (n=124); p<0.001	×	
6. Mathew, et al; 2016 [42]	Cross-sectional	India	n=190<br>M: 57<br>F: 133	≥ 60 years	MNA	OR (95% CI): single/widowed/divorced 2.189 p=0.040	○	
7. Park, et al; 2014 [27]	Cross-sectional	Korea	n=15,146<br>M: 6,185	≥ 60 years	NSI	MN: single 74.1% (n=40) p=0.001<br>Optimal nutritional status: single 25.9% (n=14) p=0.001	×	

			F: 8,961					
8. Schilp, et al; 2011 [33]	Cohort	Amsterdam	n=1,120<br>M: 577<br>F: 543	≥ 65 years	BMI	<u>HR (95% CI):</u> single 1.70 (1.24-2.33) p<0.05	×	
9. Shi, et al; 2014 [30]	Cross-sectional	China	n=558<br>M: 245<br>F: 313	≥ 60 years	MNA	MN: married 59.5% (n=75); divorced/widowed 40.5% (n=51); p=0.000		×
10. Söderhamn, et al; 2012 [34]	Cross-sectional	Norway	n=2,106<br>M: 1,043<br>F: 1,063	≥ 65 years	NUFFE-NO and MNA	<u>OR (95% CI):</u> single 2.991 (2.169-4.125) p<0.001<br><u>β coefficient:</u> single 1.096 p<0.001	○	
11. Timpini, et al; 2011 [35]	Cross-sectional	Italy	n=698<br>M: 290<br>F: 408	≥ 65 years	MNA	MN: married 5.6% (n=22); not married 11.8% (n=29); p<0.01	×	
**No significant studies**								
12. Chen, et al; 2007 [16]	Cohort	Taiwan	n=114<br>M: 50<br>F: 64	≥ 65 years	MNA	MN: widowed 22.8±4.1; married 23.6±3.0; single/divorced 24.5±4.4; p=0.38		
13. Damayanthi, et al; 2018 [29]	Cross-sectional	Sri Lanka	n=999<br>M: 251<br>F: 748	≥ 60 years	MNA	MN: married 13.8% (n=135); single 0; divorced/widowed 9.8% (n=16); p=0.095		
14. Debnath, et al; 2017 [44]	Cross-sectional	Bangladesh	n=330<br>M: 145<br>F: 185	≥ 60 years	BMI	MN: married 44.4% (n=95); single 56.4% (n=57); p=0.123		
15. Farre, et al; 2014 [26]	Cross-sectional	Spain	n=328<br>M: 126<br>F: 202	85 years	MNA	MN: married 37.2% (n=42); single 8.0% (n=9); widowed 54.9% (n=62); p=0.43		
16. Ferdous, et al; 2009 [41]	Cross-sectional	Bangladesh	n=457<br>M: 208<br>F: 249	≥ 60 years	MNA	<u>β coefficient:</u> married -0.05 p=0.434		
17. Fjell, et al; 2018 [23]	Cross-sectional	Norway	n=166<br>M: 208<br>F: 249	≥ 75 years	MNA	<u>OR (95% CI):</u> single 2.131 (0.723-6.287) p=0.170		
18. Gündüz, et al; 2015 [52]	Cross-sectional	Turkey	n=1,030<br>M: 464<br>F: 566	≥ 65 years	MNA	RMN: married 74.0% (n=222); single 1.3% (n=4); widowed 24.7% (n=74); p=0.4<br>MN: married 69.4% (n=136); single 1.0% (n=2); widowed 29.6% (n=58); p=0.4		

19. Jésus, et al; 2017 [46]	Cross-sectional	Central Africa	n=990<br>M: 406<br>F: 585	≥ 65 years	IMC	MN: single 3.2% (n=6); married 38.4% (n=73); divorced 6.3% (n=12); widowed 52.1% (n=99); p=0.89	
20. Jun, et al; 2016 [51]	Cross-sectional	Shanghai	n=2,556<br>M: 0<br>F: 2,556	≥ 60 years	MNA	MN: married 66.4% (n=704); divorced/widowed 33.6% (n=356); p=0.2840	
21. Madeira, et al; 2018 [24]	Cross-sectional	Portugal	n=1,186<br>M: 322<br>F: 864	≥ 65 years	MNA	OR (95% CI): single 1.49 (0.58-3.84); divorced/widowed 2.04 (0.93-4.48); p>0.05	
22. Maseda, et al; 2017 [13]	Cross-sectional	Spain	n=749<br>M: 295<br>F: 454	≥ 65 years	MNA	MN: single 7.5% (n=8); married 46.7% (n=50); widowed 41.1% (n=44); divorced 4.7% (n=5); p=0.064	
23. Suzana, et al; 2013 [36]	Cross-sectional	Malaysia	n=160<br>M: 58<br>F: 102	≥ 60 years	MNA	RMN: not married 46.5% (n=20) p=0.534<br>Optimal nutritional status: not married 53.5% (n=23) p=0.534	
24. Wham, et al; 2015 [48]	Cross-sectional	New Zealand	n=67<br>M: 30<br>F: 37	≥ 75 years	SCREEN II	MN: married/partner 49.1±5.37; widowed 47.0±4.83; divorced 45.4±6.43; single 47.5±9.19; p=0.34	

M: Male; F: Female; MN: Malnutrition; RMN: Risk of malnutrition; MNA: Mini Nutritional Assessment; BMI: Body Mass Index; SCREEN II: Senior in the community: risk evaluation for eating and nutrition; NSI: Nutritional Screening Initiative; NUFFE-NO: Norwegian version of Nutritional Form for the Elderly; OR: Odds Ratio; HR: Hazard Ratio; ○: Based on Odds Ratio; ×: Not based on Odds Ratio

[b]Only relation with malnutrition was established in the nursing home

Table S5. Characteristics of included studies about the relationship between income level and malnutrition and/or malnutrition risk

Study	Type of study	Country or region	Number of participants	Age	Nutritional status assessment tool	Results	Having a low-income level is related to:	
							Risk of malnutrition or malnutrition	No malnutrition
**Significant studies**								
1. Donini, et al; 2013 [14]	Cross-sectional	Italy	n=718<br>M: 246<br>F: 472	≥ 65 years	MNA	RMN nursing home: <500€ 19.7%; 501-1,000€ 45.5%; 1,001-1,500€ 27.3%; 1,500-2,000€ 7.6%; p<0.05<br>RMN home: <500€ 8.5%; 501-1,000€ 65.4%; 1,001-1,500€ 26.2%; 1,500-2,000€ 0.0%; p<0.05<br>MN nursing home: <500€ 25.4%; 501-1,000€ 57.6%; 1,001-1,500€ 15.3%; 1,500-2,000€ 1.7%; p<0.05<br>MN home: <500€ 18.9%; 501-1,000€ 75.7%; 1,001-1,500€ 5.4%; 1,500-2,000€ 0.0%; p<0.05	× [b]	
2. El-Desouky, et al; 2018 [45]	Cross-sectional	Egypt	n=320<br>M: 167<br>F: 153	≥ 60 years	MNA	<u>OR (95% CI):</u> total financial dependence 64.7 (22.7-184.5) p<0.001	○	
3. Ferdous, et al; 2009 [41]	Cross-sectional	Bangladesh	n=457<br>M: 208<br>F: 249	≥ 60 years	MNA	<u>β coefficient:</u> having income 0.14 p=0.009; receiving finance on regular basis 0.11 p=0.018	×	
4. Ferra, et al; 2012 [22]	Cross-sectional	Spain	n=402<br>M: 187<br>F: 215	≥ 65 years	BMI	<u>OR (95% CI):</u> low economic level 1.31 (0.67-2.53) p=0.001	○ [a]	
5. Jun, et al; 2016 [51]	Cross-sectional	Shanghai	n=2,556<br>M: 0<br>F: 2,556	≥ 60 years	MNA	<u>OR (95% CI):</u> high socioeconomic level 0.659 (0.511-0.876) p=0.004<br><u>β coefficient:</u> high socioeconomic level -0.347 p=0.004	○	
6. Krzymińska-Siemaszko, et al; 2014 [39]	Cross-sectional	Poland	n=4,482<br>M: 2,340<br>F: 2,142	≥ 65 years	MNA	RMN: poverty self-declaration yes 47.44% (n=222); no 41.53% (n=1,488); p<0.001<br>MN: poverty self-declaration yes 16.45% (n=77); no 9.57% (n=343); p<0.001	×	

7. Madeira, et al; 2018 [24]	Cross-sectional	Portugal	n=1,186<br>M: 322<br>F: 864	≥ 65 years	MNA	<u>OR (95% CI):</u> difficult or very difficult economic situation 3.16 (1.36-7.38) p<0.05	○	
8. Mathew, et al; 2016 [42]	Cross-sectional	India	n=190<br>M: 57<br>F: 133	≥ 60 years	MNA	<u>OR (95% CI):</u> socioeconomic classes IV, V 6.013 p=0.0000	○	
9. Mitri, et al; 2016 [25]	Cross-sectional	Lebanon	n=905<br>M: 533<br>F: 372	≥ 65 years	MNA	MN: not monthly income 55.1% (n=75); insufficient monthly income 50.8% (n=230) p=0.011<br>Optimal nutritional status: not monthly income 44.9% (n=61); insufficient monthly income 49.2% (n=223) p=0.011	×	
10. Naidoo, et al; 2015 [43]	Cross-sectional	South Africa	n=984<br>M: 224<br>F: 760	≥ 60 years	MNA	<u>OR (95% CI):</u> income <R1,600 1.425 (1.081-1.880) p=0.012<br><u>β coefficient:</u> income <R1,600 0.355 p=0.012	○	
11. Olayiwola, et al; 2006 [47]	Cross-sectional	Nigeria	n=305<br>M: 163<br>F: 142	≥ 60 years	NSI	<u>Pearson correlation</u>: having income r=0.31		×
12. Park, et al; 2014 [27]	Cross-sectional	Korea	n=15,146<br>M: 6,185<br>F: 8,961	≥ 60 years	NSI	MN: monthly income (1,000 US$) <0.5: 67.9% (n=2,873) p=0.001<br>Optimal nutritional status: monthly income (1,000 US$) <0.5: 32.1% (n=1,356) p=0.001	×	
13. Timpini, et al; 2011 [35]	Cross-sectional	Italy	n=698<br>M: 290<br>F: 408	≥ 65 years	MNA	<u>OR (95% CI):</u> bad economic level 2.4 (1.1-5.3) p<0.05	○	
**No significant studies**								
14. Chen, et al; 2007 [16]	Cohort	Taiwan	n=114<br>M: 50<br>F: 64	≥ 65 years	MNA	MN: monthly income <5,000 NTD 23.4±2.4; 5,000-1,000 23.4±2.3; >10,001-20,000 23.2±4.6; prefer not to tell 23.5±3.5; p=0.99		
15. Damayanthi, et al; 2018 [29]	Cross-sectional	Sri Lanka	n=999<br>M: 251<br>F: 748	≥ 60 years	MNA	<u>OR (95% CI):</u> below poverty line RMN 1.29 (0.69-2.41) p=0.400; MN 1.05 (0.45-2.44) p=0.909		
16. Debnath, et al; 2017 [44]	Cross-sectional	Bangladesh	n=330<br>M: 145<br>F: 185	≥ 60 years	BMI	MN: monthly income <10,000 BDT 53.5% (n=23); 10,001-20,000 BDT 52.3% (n=81) ; 20,001 BDT 41.0% (n=48); p=0.058		
17. El Zoghbi, et al; 2014 [20]	Cross-sectional	Lebanon	n=111<br>M: 55<br>F: 56	≥ 65 years	MNA	RMN: <200$ 62.8% (n=27); 200-400 25.6% (n=11); >400 11.6% (n=5); p=0.915<br>MN: <200$ 80.0% (n=8); 200-400 20.0% (n=2); >400 0.0% (n=0); p=0.915		

18. Ji, et al; 2012 [37]	Cross-sectional	China	n=632<br>M: 208<br>F: 424	≥ 90 years	MNA	RMN: income level <1,000 RMB/year 42.9% p=0.36<br>MN: income level <1,000 RMB/year 52.8% p=0.36	
19. Schilp, et al; 2011 [33]	Cohort	Amsterdam	n=1,120<br>M: 577<br>F: 543	≥ 65 years	BMI	HR (95% CI): no income 0.93 (0.54-1.62) p>0.05	
20. Shi, et al; 2014 [30]	Cross-sectional	China	n=558<br>M: 245<br>F: 313	≥ 60 years	MNA	MN: monthly income 1663.35±149.98 p=0.177	

M: Male; F: Female; MN: Malnutrition; RMN: Risk of malnutrition; MNA: Mini Nutritional Assessment; BMI: Body Mass Index; NSI: Nutritional Screening Initiative; R: Rand (South African currency); RMB: Renminbi (Chinese currency); NTD: New Taiwan Dollar (Taiwanese currency); BDT: Taka (Bangladesh currency); OR: Odds Ratio; HR: Hazard Ratio; ○: Based on Odds Ratio; ×: Not based on Odds Ratio

Socioeconomic classes: IV, 870-1,730 income per capita; V, <870 income per capita

[a]Odds Ratio obtained from the low weight evaluated by the Body Mass Index; [b]Only relation with malnutrition was established in the nursing home

Table S6. Characteristics of included studies about the relationship between occupational level and malnutrition and/or malnutrition risk

Study	Type of study	Country or region	Number of participants	Age	Nutritional status assessment tool	Results	Having a low occupational level is related to:	
							Risk of malnutrition or malnutrition	No mal-nutrition
**Significant studies**								
1. Donini, et al; 2013 [14]	Cross-sectional	Italy	n=718<br>M: 246<br>F: 472	≥ 65 years	MNA	RMN nursing home: housewife 16.2%; employee 13.2%; worker 11.8%; retired 58.8%; p<0.05<br>RMN home: housewife 23.8%; employee 6.2%; worker 3.1%; retired 66.9%; p<0.05<br>MN nursing home: housewife 17.2%; employee 9.4%; worker 6.3%; retired 67.2%; p<0.05<br>MN home: housewife 21.6%; employee 0.0%; worker 0.0%; retired 78.4%; p<0.05	×	
2. El-Desouky, et al; 2018 [45]	Cross-sectional	Egypt	n=320<br>M: 167<br>F: 153	≥ 60 years	MNA	<u>OR (95% CI):</u> unemployed 22.2 (11.8-41.7) p<0.001	○	
3. Jésus, et al; 2017 [46]	Cross-sectional	Central Africa	n=990<br>M: 406<br>F: 585	≥ 65 years	BMI	<u>OR (95% CI):</u> farmer/breeder 1.8 (1.2-2.7) p=0.006[c]	○	
4. Lin, et al; 2016 [53]	Cross-sectional	China	n=708<br>M: 377<br>F: 371	≥ 60 years	MNA	MN: retired 19.6% (n=32); not retired 27.5% (n=150); p=0.043		×
5. Mokhber, et al; 2011 [28]	Cross-sectional	Iran	n=1,565<br>M: 720<br>F: 845	≥ 60 years	MNA	MN: self-employed 5%; employee 6.3%; farmer 9.5%; worker/laborer 9.6%; unemployed 12.7%; p=0.001	×	
6. Söderhamn, et al; 2012 [34]	Cross-sectional	Norway	n=2,106<br>M: 1,043<br>F: 1,063	≥ 65 years	NUFFE-NO and MNA	<u>OR (95% CI):</u> administration professions 0.497 (0.361-0.685) p<0.001<br><u>β coefficient:</u> administration professions -0.699 p<0.001	○	
**No significant studies**								
7. Debnath, et al; 2017 [44]	Cross-sectional	Bangladesh	n=330<br>M: 145<br>F: 185	≥ 60 years	BMI	MN: unemployed 46.9% (n=120); employee 54.2% (n=32); p=0.779		

8. Ji, et al; 2012 [37]	Cross-sectional	China	n=632<br>M: 208<br>F: 424	≥ 90 years	MNA	RMN: farmers 80.8% p=0.39<br>MN: farmers 83.3% p=0.39	
9. Jun, et al; 2016 [51]	Cross-sectional	Shanghai	n=2,556<br>M: 0<br>F: 2,556	≥ 60 years	MNA	MN: hard manual work 52.5% (n=556); light physical work 31.7% (n=336); mental effort 15.8% (n=168); p=0.8350[d]	
10. Shi, et al; 2014 [30]	Cross-sectional	China	n=558<br>M: 245<br>F: 313	≥ 60 years	MNA	MN: manual work 9.5% (n=12); moderate physical work 34.9% (n=44); mental effort 55.6% (n=70); p=0.462[d]	
11. Suzana, et al; 2013 [36]	Cross-sectional	Malaysia	n=160<br>M: 58<br>F: 102	≥ 60 years	MNA	RMN: working 35.7% (n=10); not working 43.9% (n=58); p=0.424	
12. Timpini, et al; 2011 [35]	Cross-sectional	Italy	n=698<br>M: 290<br>F: 408	≥ 65 years	MNA	MN: not manual work 6.4% (n=16); manual work 9.1% (n=34); p=0.216[d]	

M: Male; F: Female; MN: Malnutrition; RMN: Risk of malnutrition; MNA: Mini Nutritional Assessment; BMI: Body Mass Index; NUFFE-NO: Norwegian version of Nutritional Form for the Elderly; OR: Odds Ratio; ○: Based on Odds Ratio; ×: Not based on Odds Ratio

[c]Previous (before 60 years old) and current occupational level; [d]Only previous (before 60 years old) occupational level

Table S7. Characteristics of included studies about the relationship between feeling of loneliness and malnutrition and/or malnutrition risk

Study	Type of study	Country or region	Number of participants	Age	Nutritional status assessment tool	Results	Having feeling of loneliness is related to:	
							Risk of malnutrition or malnutrition	No mal-nutrition
**Significant studies**								
1. Boulos, et al; 2016 [17]	Cross-sectional	Lebanon	n=1,020<br>M: 515<br>F: 505	≥ 65 years	MNA	RMN: Wilson score 0-1, 20.1%; 2-3, 41.8%; 4-5, 46.0%; p<0.001<br>MN: Wilson score 0-1, 1.9%; 2-3, 5.1%; 4-5, 25.0%; p<0.001<br>Optimal nutritional status: Wilson score 0-1, 78.0%; 2-3, 53.1%; 4-5, 29.0%; p<0.001	×	
2. Eskelinen, et al; 2016 [21]	Cross-sectional	Finland	n=573<br>M: 171<br>F: 402	≥ 75 years	MNA	MN: feeling of loneliness often 43.8% (n=81); never 56.2% (n=104); p<0.001		×
3. Maseda, et al; 2017 [13]	Cross-sectional	Spain	n=749<br>M: 295<br>F: 454	≥ 65 years	MNA	MN: feeling of loneliness often 7.5% (n=8); sometimes 25.2% (n=27); hardly ever 67.3% (n=72); p=0.028		×
4. Schilp, et al; 2011 [33]	Cohort	Amsterdam	n=1,120<br>M: 577<br>F: 543	≥ 65 years	IMC	HR (95% CI): loneliness 1.47 (1.06-2.04) p<0.05	×	
**No significant studies**								
5. Söderhamn, et al; 2012 [34]	Cross-sectional	Norway	n=2,106<br>M: 1,043<br>F: 1,063	≥ 65 years	NUFFE-NO and MNA	OR (95% CI): loneliness 1.611 (0.987-2.629) p=0.056<br>β coefficient: loneliness 0.477 p=0.056		
6. Suzana, et al; 2013 [36]	Cross-sectional	Malaysia	n=160<br>M: 58<br>F: 102	≥ 60 years	MNA	RMN: not loneliness 46.2% (n=12); loneliness 41.8% (n=56); p=0.680		

M: Male; F: Female; MN: Malnutrition; RMN: Risk of malnutrition; MNA: Mini Nutritional Assessment; BMI: Body Mass Index; NUFFE-NO: Norwegian version of Nutritional Form for the Elderly; OR: Odds Ratio; HR: Hazard Ratio; ○: Based on Odds Ratio; ×: Not based on Odds Ratio

Wilson score: 0-1, no feeling of loneliness; 2-3, moderate feeling of loneliness; 4-5, important feeling of loneliness

Table S8. Characteristics of included studies about the relationship between place of residence and malnutrition and/or malnutrition risk

Study	Type of study	Country or region	Number of participants	Age	Nutritional status assessment tool	Results	Living in rural areas is related to:	
							Risk of malnutrition or malnutrition	No mal-nutrition
**Significant studies**								
1. Mokhber, et al; 2011 [28]	Cross-sectional	Iran	n=1,565<br>M: 720<br>F: 845	≥ 60 years	MNA	RMN: rural 47.8%; urban 41.7%; p=0.001<br>MN: rural 13.1%; urban 10.5%; p=0.001	×	
**No significant studies**								
2. Jun, et al; 2016 [51]	Cross-sectional	Shanghai	n=2,556<br>M: 0<br>F: 2,556	≥ 60 years	MNA	MN: urban 62.5% (n=662); rural 37.5% (n=398); p=0.3820		
3. Krzymińska-Siemaszko, et al; 2014 [39]	Cross-sectional	Poland	n=4,482<br>M: 2,340<br>F: 2,142	≥ 65 years	MNA	RMN: rural 43.94% (n=772); city 41.76% (n=1,138); p=UA<br>MN: rural 12.64% (n=222); city 10.83% (n=295); p=UA		

M: Male; F: Female; MN: Malnutrition; RMN: Risk of malnutrition; MNA: Mini Nutritional Assessment; BMI: Body Mass Index; NUFFE-NO: Norwegian version of Nutritional Form for the Elderly; UA: Unavailable; ○: Based on Odds Ratio; ×: Not based on Odds Ratio

Table S9. Characteristics of included studies about the relationship between food expenditure and malnutrition and/or malnutrition risk

Study	Type of study	Country or region	Number of participants	Age	Nutritional status assessment tool	Results	Low food expenditure is related to: Risk of malnutrition or malnutrition	No mal-nutrition
**No significant studies**								
1. Ferdous, et al; 2009 [41]	Cross-sectional	Bangladesh	n=457<br>M: 208<br>F: 249	≥ 60 years	MNA	β coefficient: daily food expenditure 0.01 p=0.746		

M: Male; F: Female; MNA: Mini Nutritional Assessment

Table S10. Quality assessment of the observational studies included in systematic review

	1. Research question	2. Study population	3. Participation rate	4. Population selection	5. Sample size justification	6. Exposure assessment	7. Sufficient timeframe to see and effect	8. Different levels of exposure	9. Exposure measures and assessment	10. Repeated exposure assessment	11. Outcome measures	12. Blinding of outcomes assessors	13. Follow-up rate	14. Statistical analyses	Quality:
1. Bardon, et al; 2018 [19]	✓	✓	✓	✓	×	×	NR	✓	✓	×	✓	NR	✓	✓	Medium
2. Boulos, et al; 2016 [17]	✓	✓	✓	✓	×	×	×	✓	✓	×	✓	NA	NA	✓	High
3. Cabrera, et al; 2007 [18]	✓	✓	✓	✓	×	×	×	×	NR	×	✓	NA	NA	✓	Medium
4. Chen, et al; 2007 [16]	✓	✓	✓	✓	×	NR	NR	✓	✓	×	✓	NR	NR	✓	Medium
5. Damayanthi, et al; 2018 [29]	✓	✓	✓	✓	✓	×	×	✓	✓	×	✓	NA	NA	✓	High
6. Debnath, et al; 2017 [44]	✓	✓	✓	✓	✓	×	×	✓	×	×	✓	NA	NA	×	Medium
7. Donini, et al; 2013 [14]	✓	×	NA	×	×	×	×	✓	✓	×	✓	NA	NA	×	Medium
8. El Zoghbi, et al; 2014 [20]	✓	✓	×	✓	×	×	×	✓	✓	×	✓	NA	NA	✓	Medium
9. El-Desouky, et al; 2018 [45]	✓	✓	NA	✓	✓	×	×	✓	✓	×	✓	NA	NA	✓	High
10. Eskelinen, et al; 2016 [21]	×	✓	NA	NR	×	×	×	NA	✓	×	✓	NA	NA	✓	Medium
11. Farre, et al; 2014 [26]	✓	✓	✓	✓	×	×	×	✓	✓	×	✓	NA	NA	✓	High
12. Ferdous, et al; 2009 [41]	✓	✓	✓	NR	✓	×	×	✓	✓	×	✓	NA	NA	✓	High
13. Ferra, et al; 2012 [22]	✓	✓	✓	✓	✓	×	×	✓	✓	×	✓	NA	NA	✓	High
14. Fjell, et al; 2018 [23]	✓	✓	✓	✓	×	×	×	NR	✓	×	✓	NA	NA	✓	Medium
15. Gündüz, et al; 2015 [52]	✓	✓	NA	NR	×	×	×	✓	✓	×	✓	NA	NA	✓	Medium
16. Jésus, et al; 2017 [46]	✓	✓	✓	✓	×	×	×	✓	✓	×	✓	NA	NA	✓	High
17. Ji, et al; 2012 [37]	✓	✓	✓	✓	×	×	×	NA	✓	×	✓	NA	NA	✓	Medium
18. Johansson, et al; 2009 [40]	✓	×	✓	×	×	×	×	NA	✓	✓	✓	NA	NA	✓	Medium
19. Jun, et al; 2016 [51]	✓	✓	✓	✓	×	×	×	✓	✓	×	✓	NA	NA	✓	High
20. Krzymińska-Siemaszko, et al; 2014 [39]	✓	✓	✓	✓	✓	×	×	NA	NR	×	✓	NA	NA	✓	Medium
21. Lengyel, et al; 2017 [50]	✓	✓	✓	✓	×	✓	NR	NR	×	✓	✓	NR	NR	×	Medium

22. Lin, et al; 2016 [53]	✓	✓	✓	✓	×	×	×	✓	✓	×	✓	NA	NA	✓	High
23. Madeira, et al; 2018 [24]	✓	✓	✓	✓	×	×	×	✓	✓	×	✓	NA	NA	✓	High
24. Maseda, et al; 2017 [13]	✓	✓	NA	✓	×	×	×	✓	✓	×	✓	NA	NA	✓	Medium
25. Mathew, et al; 2017 [42]	✓	✓	NA	✓	✓	×	×	NA	✓	×	✓	NA	NA	✓	Medium
26. Mitri, et al; 2016 [25]	✓	✓	NA	✓	✓	×	×	✓	✓	×	✓	NA	NA	✓	High
27. Mokhber, et al; 2011 [28]	✓	×	✓	NR	×	×	×	NA	×	×	✓	NA	NA	×	Low
28. Naidoo, et al; 2015 [43]	✓	✓	✓	✓	✓	×	×	✓	✓	×	✓	NA	NA	✓	High
29. Olayiwola, et al; 2006 [47]	✓	✓	NA	✓	×	×	×	✓	×	×	✓	NA	NA	✓	Medium
30. Park, et al; 2014 [27]	✓	✓	NA	NR	×	×	×	✓	NR	×	✓	NA	NA	×	Medium
31. Ramage-Morin, et al; 2013 [49]	✓	✓	✓	✓	×	×	×	NA	✓	×	✓	NA	NA	✓	Medium
32. Rodriguez-Tadeo, et al; 2011 [32]	✓	✓	NA	×	×	×	×	✓	✓	×	✓	NA	NA	✓	Medium
33. Romero-Ortuno, et al; 2010 [31]	✓	✓	NA	NR	×	×	×	×	✓	×	✓	NA	NA	✓	Medium
34. Schilp, et al; 2011 [33]	✓	✓	NA	✓	×	✓	✓	✓	✓	✓	✓	NR	NR	✓	High
35. Shi, et al; 2014 [30]	✓	✓	✓	✓	×	×	×	✓	✓	×	✓	NA	NA	✓	High
36. Söderhamn, et al; 2012 [34]	✓	✓	NA	NR	×	×	×	✓	✓	×	✓	NA	NA	✓	Medium
37. Suzana, et al; 2013 [36]	✓	✓	NA	✓	×	×	×	NA	✓	×	✓	NA	NA	✓	Medium
38. Timpini, et al; 2011 [35]	✓	✓	✓	NR	×	×	×	NA	✓	×	✓	NA	NA	✓	Medium
39. Westergren, et al; 2014 [38]	✓	✓	✓	✓	×	×	×	✓	✓	×	✓	NA	NA	✓	High
40. Wham, et al; 2015 [48]	✓	✓	✓	✓	×	×	×	✓	✓	×	✓	NA	NA	✓	High
**TOTAL OF AFFIRMATIVE RESULTS**	**39**	**37**	**25**	**29**	**9**	**2**	**1**	**27**	**33**	**3**	**40**	**0**	**1**	**35**	

NA: Not applicable; NR: Not reported
